# A rich dataset of hourly residential electricity consumption data and survey answers from the iFlex dynamic pricing experiment

**DOI:** 10.1016/j.dib.2023.109571

**Published:** 2023-09-15

**Authors:** Matthias Hofmann, Turid Siebenbrunner

**Affiliations:** a Statnett SF, Nydalen allé 33, 0484 Oslo, Norway; b Department of Electric Power Engineering, NTNU, O. S. Bragstads plass 2E, 7034 Trondheim, Norway

**Keywords:** Demand-side flexibility, Demand response, Price elasticity, Panel data, Real-time pricing, Households

## Abstract

The iFlex field experiment was conducted to understand if and how households change their power consumption in response to variable hourly electricity prices. This experiment was conducted in several Norwegian regions, and various price signals were tested over two winter periods from early 2020 to spring 2021. The resulting dataset includes hourly electricity consumption data of all participating households and answers to three surveys about household characteristics such as electric appliances, living conditions, socio-demographic variables, and their willingness to be flexible. In addition, temperature data are added to the dataset from public sources. This rich dataset can be used to analyse households' demand flexibility potential in-depth. Furthermore, subgroups, such as low-income households or highly electrified households with electricity as a primary heating source, can be investigated to enhance the understanding of how these are affected by variable power prices.

Specifications Table

**Table:** 

Subject	Energy Economics
Specific subject area	Dynamic pricing experiment to reveal demand-side flexibility of residential electricity consumers in response to variable hourly power prices
Type of data	Table (csv files and RData file)
How the data were acquired	Electricity consumption: Smart meter measurements Household characteristics: Telephone and web surveys Outdoor temperature: observations from weather stations of the Norwegian Meteorological Institute All data were processed with R, a free software environment for statistical computing and graphics [1]
Data format	Raw Analysed Filtered
Description of data collection	Electricity consumption data of Norwegian households were collected upfront and during the iFlex price experiment for two winter seasons in early 2020 and 2020/21. Households had to opt into the project and consented to share their data from the national database for meter data Elhub or their electricity suppliers. Three surveys were conducted by telephone and online with participating households and one with a household panel representing the general population. Outdoor temperature: Public available hourly temperature series from the Norwegian Meteorological Institute for the same period as the electricity consumption data.
Data source location	Electricity consumption and survey answers (primary data): • Regions: Oslo, Stavanger, Bergen, Trondheim, Bodø, Tromsø • Country: Norway Outdoor temperature (secondary data): • Data source: Norwegian Meteorological Institute (https://seklima.met.no/observations/) • Weather stations (latitude, longitude): ○ Oslo – Blindern (59.9423° N, 10.720° E) ○ Stavanger – Våland (58.9563° N, 5.7278° E) ○ Bergen – Florida (60.3830° N, 5.3327° E) ○ Trondheim – Risvollan (63.3987° N, 10.4228° E) ○ Bodø – Skivika (67.3084° N, 14.4309° E) ○ Tromsø (69.6537° N, 18.9368° E) • Country: Norway
Data accessibility	Repository name: Zenodo Data identification number: 10.5281/zenodo.8248802 Direct URL to data: https://zenodo.org/record/8248802

## 1 Value of the Data

• Demand response from residential electricity consumers to variable power prices will play an important role in the future carbon-neutral energy system, and this dataset gives a unique possibility to study the potential of demand-side flexibility from households.
• The dataset is rich since it combines electricity consumption data from a pricing experiment for an extensive range of price signals with survey data, including household characteristics such as electric appliances, living conditions, socio-demographic variables, and willingness to be flexible.
• Most participating households are highly electrified (electric cars, electrified heating, and electric boilers for hot water). They are of interest since all households may need to switch to electricity as the primary energy source in the future to slow climate change.
• The dataset is useful for professionals, researchers and students in electricity economics, behavioural economics, or social science who are interested in understanding the relationship between different types of households, their electricity consumption, and their ability to respond to variable electricity prices. Teachers of economics or statistics courses may use the data to learn basic statistics as for example hypothesis testing or regression analysis with software such as R, Python, SPSS, Stata and others.
• Based on the data, further insight can be gained for improving electricity consumption prognosis models and the performance testing of demand baseline calculation methods.
• The analysis of different household subgroups and inequalities between them, for example, vulnerable households and their ability to be flexible and gain from variable power prices, can be investigated with the data.

## 2 Objective

Statnett, the Norwegian transmission system operator, conducted the iFlex project to understand how variable end-user electricity prices change the power demand in peak hours. The objective was to establish estimates for price sensitivity, which could be used for improving long-term demand prognosis and could be included in power market models. Furthermore, the results should quantify the potential of demand flexibility triggered by price signals, also known as implicit demand flexibility.

The dataset was used in two scientific articles [2,3], and its publication enriches them by adding a detailed description of the data collection process so that other researchers can critically review the data quality that led to the results in these articles.

## 3 Data Description

**Fig. 1:**
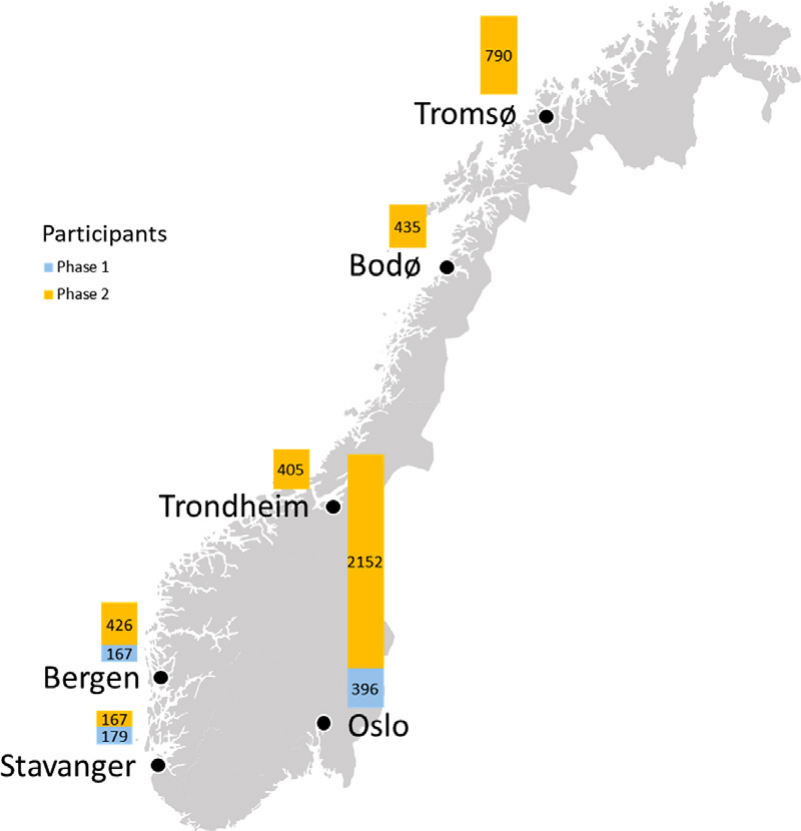
Location of the city regions with the number of participating households in the pricing experiment, i.e., available electricity consumption data.

A field experiment with Norwegian households was conducted by testing their response to artificial prices for electricity that change hourly on some experiment days. This dataset contains the data which were collected during the experiment. As shown in Fig. 1, households from six city regions participated in the experiment, and the data collection was carried out in two phases: the first in winter 2019/20 and the second in winter 2020/21. Phase 1 was a pilot with considerably fewer participating households than the full-scale pricing experiments in Phase 2. The main focus of the pilot in Phase 1 was testing the experimental setup and the practical conduction of the pricing experiments while collecting data for further analysis. First, we describe the different types of data, and second, the structure of the table files.

### 3.1 Collected type of data

**Fig. 2:**
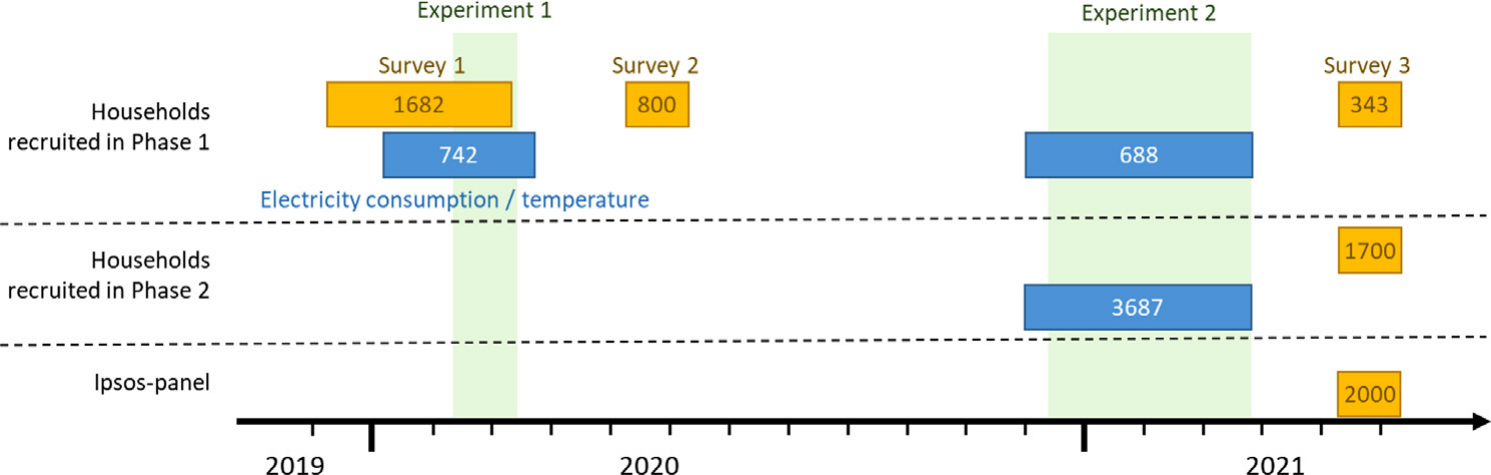
Overview of the periods when the surveys were conducted, and data have been collected and for how many households these data are available (number in the boxes) dependent on when they have been recruited.

The pricing experiment is specified by the variables of participating households in the various control and treatment groups and the price signals. Furthermore, two different types of data were collected directly by the project, i.e., hourly electricity consumption and survey answers. In addition, the data collection was complemented with outdoor temperature data from the Norwegian Meteorological Institute since electricity consumption in Norwegian households is highly dependent on outdoor temperature due to the widespread use of electrical heating. All these data types are shortly described in the following sub-chapters. Fig. 2 shows when and from how many households data were collected. Not all households participating in the pricing experiment answered a survey, and those answering Survey 1 did not necessarily share their electricity consumption data. Therefore, the richness of the data is different per household, e.g., households answering all surveys and participating in both project phases, thus sharing their electricity consumption versus households only participating in Phase 2, which may not have answered Survey 3.

### 3.2 Experiment variables

Participating households were located in one of six regions and either in a control or treatment group. Table 1 summarises the final number of households per group and region in the dataset and which project partner was responsible for sending them information about the price signals.

**Table 1:** Control (indicated by ‘Control’ in the group name) and treatment groups per project phase and region based on information from participants.csv.

Project phase	Responsible	Group name	Number of households						
			Sum	Oslo	Sta-vanger	Bergen	Trond-heim	Bodø	Tromsø
*Phase 1*			*742*	*396*	*179*	*167*			
	Transmission system operator	Control	303	171	68	64			
		H1	219	110	55	54			
		H2	220	115	56	49			
*Phase 2*			*3,741*	*2,152*	*167*	*426*	*405*	*435*	*790*
	Transmission system operator	Control	279	157	63	59			
		H1	203	100	52	51			
		H2	206	108	52	46			
	Electricity supplier 1	Os_Control	257	257					
		Os_1	255	255					
		Os_2	247	247					
		Os_3	254	254					
		Os_4	261	261					
		Os_5	262	262					
		Os_6	251	251					
		Ber_Control	52			52			
		Ber_1	218			218			
		Trond_Control	204				204		
		Trond_1	201				201		
	Electricity supplier 2	Trom_Control	266						266
		Trom_1	259						259
		Trom_2	265						265
	Electricity supplier 3	Bo_Control	214					214	
		Bo_1	221					221	

**Fig. 3:**
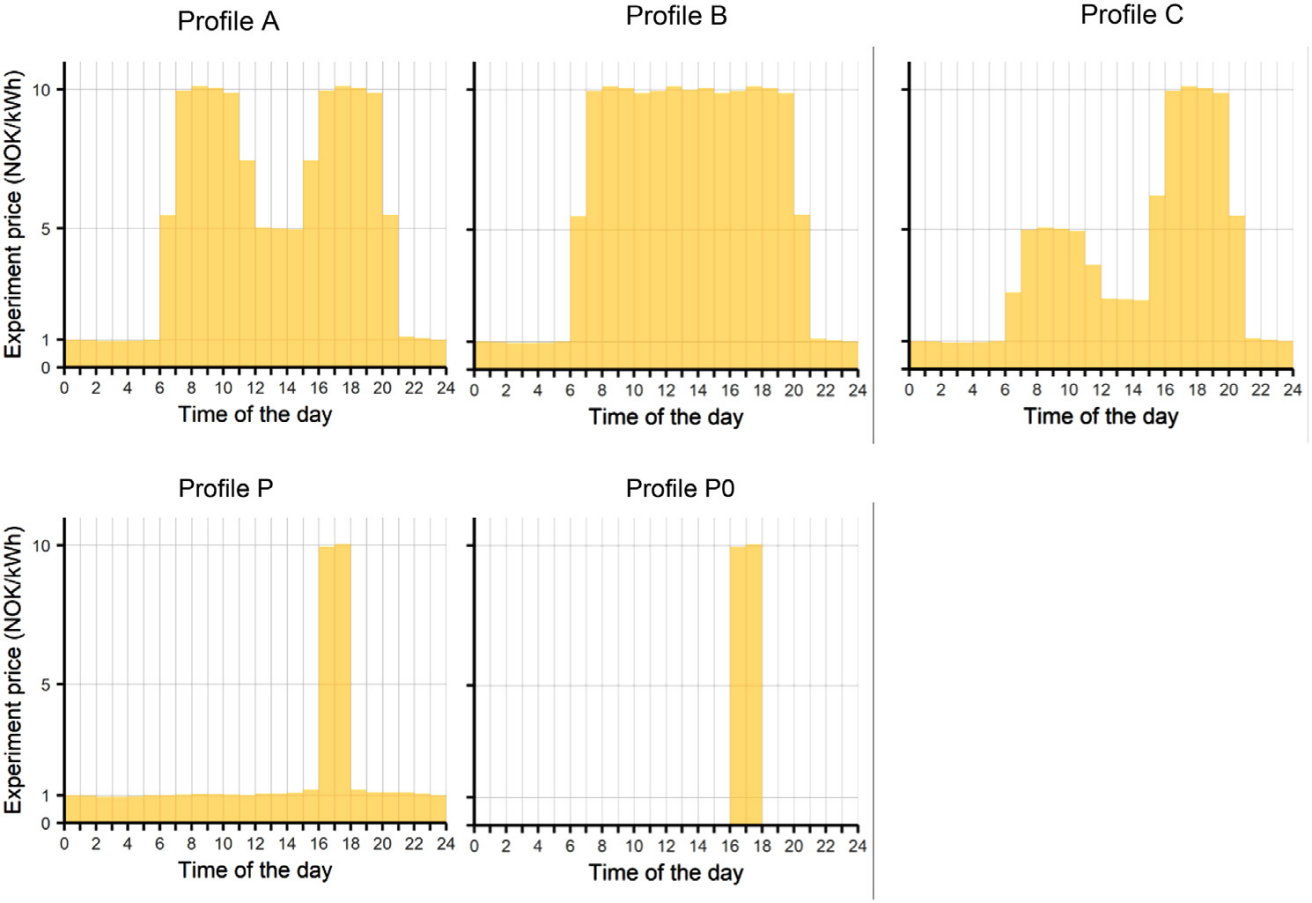
Different price profiles are exemplified with a price level of 10 NOK/kWh.

Price signals were defined as hourly electricity prices over one day. Different price signals were used in the experiment, and they consisted of five price levels, 2, 5, 10, 15, and 30 NOK/kWh, and five price profiles, A, B, C, P, and P_0. A price level equals the peak prices, and the profile describes when the peak prices occurred during the day. Minor hourly variations around the price levels have been introduced to test automatic response based on control algorithms. However, in the end, the pricing experiment tested only manual response, and these minor variations should have no practical consequence. Fig. 3 gives an overview of the different price profiles visualised with the same price level of 10 NOK/kWh. Profile A has a peak price period in the morning and afternoon, while profile B has peak prices for all daytime hours. Profile C is similar to profile A but has higher prices in the afternoon than in the morning. In profile P, the peak price duration is two hours in the afternoon, and the only difference to profile P0 is that the prices are not zero in the low-price period. The price profiles were defined to reflect Norway's average daily demand profile and typical spot price profiles.

Not all combinations of price profiles and price levels were included in the experiment due to a limited number of treatment groups and experiment days. Table 2 gives an overview of all price signals and the respective combinations of price levels and profiles used during the pricing experiment.

**Table 2:** Specification of the tested price signals as a combination of the price level (x-axis) and price profile (y-axis). For example, price profile P was tested with peak prices of 2, 5, 10 and 15 NOK/kWh.

Price level (NOK/kWh)	Price profile				
	A	B	C	P	P0
2		B_2		P_2	P0_2
5	A_5	B_5	C*	P_5	
10	A_10	B_10	C*	P_10	P0_10
15		B_15		P_15	
30					P0_30

⁎ Profile C is one price signal with different prices in morning and afternoon peak hours

The number of days on which the different price signals were tested is not equal since they could be used in a different total number of treatment groups. In addition, the experiment period was significantly shorter in Phase 1, resulting in mainly two repetitions of the price signals, whereas it was possible to test a price signal around five times per treatment group in Phase 2. Table 3 summarises how many days a price signal was used during the two experiment winters.

**Table 3:** The period and number of days on which the price signals were tested.

	Phase 1	Phase 2
First experiment day	11.02.2020	16.12.2020
Last experiment day	09.03.2020	25.03.2021
		
Price signal		
A_5	2	9
A_10	2	10
B_2		35
B_5	1	29
B_10	1	39
B_15		28
C	2	8
P_2		16
P_5	2	13
P_10	3	27
P_15		20
P0_2		5
P0_10		10
P0_30		5

### 3.3 Electricity consumption

Hourly electricity consumption data were collected mainly during the pricing experiments and for a short period before and after the experiment period. In Phase 1, the experiment was stopped early due to the start of the Covid-19 pandemic. Table 4 summarises the collection period and the consumption data available in the dataset.

**Table 4:** Summary information of the hourly electricity consumption data.

	Phase 1	Phase 2
First day	06.01.2020	01.12.2020
Last day	20.03.2020	26.03.2021
Households	742	4,375
Hours	1,800	2,784
Observations	1,335,600	12,180,000

### 3.4 Household surveys

Three surveys were conducted during the project. In Phase 1, two surveys were used to get background information about the participating households. Survey 1 collected information about demographics, housing characteristics, and electricity-consuming equipment before the experiment started. After the pricing experiments of Phase 1 were finished, Survey 2 asked if households were willing to respond to price signals, the needed economic incentive and other motivations. In addition, the households in the treatment groups were asked if and how they responded to the price signals.

In Phase 2, the third survey contained a combination of questions from Surveys 1 and 2. This survey was also sent to households that were not part of the project but lived in the same cities to compare the survey answers of the project participants with the general population and to increase the knowledge about the external validity of the pricing experiment by revealing possible biases. Table 5 summarises some main characteristics of the surveys.

**Table 5:** Information about the conducted surveys.

	Project phase	Conduction period	Population	Number of answers
Survey 1	Phase 1	10 December 2019 - 10 March 2020	Participants Phase 1	1682
Survey 2	Phase 1	12 May - 9 June 2020	Participants Phase 1	800
Survey 3 - Participants	Phase 2	12 May - 10 June 2021	Participants Phase 2	2043
Survey 3 – Norway	Phase 2	12 May - 10 June 2021	Pre-recruited Ipsos-panel	2000

The questions in the different surveys are provided below to give an overview of the household information gathered. The detailed questions and corresponding answer options can be found in the survey question files as described in section 3.2.5.

Table 6.

**Table 6:** List of questions which were used in the three surveys.

Survey 1
In this project, we want to talk with the person in the household who receives the electricity bill - are you the recipient of the electricity bill? What type of electricity contract do you have? Do you buy electricity that is guaranteed to be renewable? What type of building do you live in? How large is the home in square meters? Do you own the home? Do you have a rental unit in the home? Does the rental unit have a separate electricity meter? Do several tenants live in the home and share the flat? What year was the building you are answering for built? Has the home been renovated to reduce energy consumption? How is the home heated? In the home you are answering for, how often do you use the fireplace/wood stove? What indoor temperature do you usually prefer in the living room when you want to feel comfortable? Temperature: Do you have a lower indoor temperature in rooms that are not used that often? Do you reduce the temperature at night or when no one is home? Do you control the heating? How is the hot water heated in your home? Do you actively control the electric water heater by switching it on and off or changing the temperature? What air ventilation system do you have in your home? Do you actively follow your own electricity consumption? How do you get information about your electricity consumption? Do you actively follow how electricity prices vary? How do you get information about electricity prices? Do you have a car in your household? How many cars do you have in your household? Do you have an electric car or plug-in hybrid in your household? How many electric cars or plug-in hybrid cars does your household have? What is the maximum range in kilometres the car can drive electrically? Where is the car usually charged? How is the car charged at home? How often do you charge your car at home? At what time do you usually charge your car? Do you control the charging? How far, in terms of kilometres, do you usually drive the car before charging it? Do you have solar cells? How large is the photovoltaic system? Size in kilowatts (kW): Do you have a battery (not an electric car) connected to the photovoltaic system? Is electricity usage from a farm or business activity included in the same electricity meter? Sex? How many people, yourself included, live in your household? How old are the household residents? What is the highest completed education in the household? What is the total gross income before taxes of the household? Is someone usually at home during the day on weekdays? In what life situation are the household residents? (kindergarten, student, part-time or full-time job, unemployed, retired, other) Which of the following alternatives describes your household? (living alone, couple with or without children, single adult with children, multi-family, other)

Survey 2

Have there been any major changes in your household that may have affected your electricity consumption during February this year? Imagine that you undertake measures to reduce your electricity consumption for 4 consecutive hours some days in winter when it is cold outside, and you are at home. What consequences do you think the following measures would have for you? Could any of the following motivate you to reduce your electricity consumption for 4 consecutive hours some days in winter when it is cold outside, and you are at home?

**Table 6:** (*continued*)

Survey 2
Why do you not want to reduce your electricity consumption? If you were to change your electricity consumption pattern, how likely is it that you would do the following ...? Approximately how large total electricity expenses (incl. all taxes, grid fees, etc.) had your household on average per month during the last year? Approximately how much did your household pay per kWh of electricity (incl. all taxes, grid fees, etc.) on average during the last year? How much money do you need to save in one day to be interested in reducing your electricity consumption by 10% for 4 consecutive hours with high electricity prices on a winter day? How much money do you need to save per month, to be interested in reducing your electricity consumption by 10% for 4 consecutive hours per day, 10 days in the winter months? How much money do you need to save per month, to be interested in reducing your electricity consumption by 10% for 4 consecutive hours on all weekdays during the winter months? How much money do you need to save per month if you could switch to a cheaper electricity supplier? Imagine that you could get paid to reduce your electricity consumption. How much money do you need to earn per month, to be interested in reducing your electricity consumption by 10% for 4 consecutive hours per day, 10 days in the winter months? How much money do you need to save per month on average in the next few years in order to think about buying electricity-saving equipment that costs NOK 5,000? The equipment will reduce your electricity bill by automatically controlling parts of your consumption during the day without inconvenience to you. Would you use a free information service that notifies you when the price of electricity the next day would be over a given level for a few hours? How much higher has the electricity price to be compared to an average electricity price of 1 NOK/kWh so that you want to be notified? We want to find out how information about electricity costs can best be communicated to households. Which of the following formulations/illustrations gives you the best opportunity to understand how much you can save at different times? Your household participated in Statnett's price experiment in February and March 2020. We are interested in how you experienced the experiment and have, therefore, some questions. Did you actively follow the price signals given during the experiment? Did you undertake measures in your household to reduce or shift electricity consumption during hours with high prices? What measures did you undertake? Why did you not undertake such measures? Why did you not actively follow the price signals given during the experiment?

Survey 3

Sex? Age? Are you responsible for paying the electricity bill? How many people, yourself included, usually lived in your household in the period January - March 2021? In what age group (s) are the household residents? What type of building do you live in? How large is the home in square meters? Do you own the home? Do you have a rental unit in the home? Does the rental unit has a separate electricity meter? How is the home heated? How is the hot water heated in your home? Do you have an electric car(s) in the household that is occasionally charged at home? How is the electric car(s) usually charged? Do you control the charging of the electric car(s) to avoid hours of high electricity prices? What type of electricity contract do you have? What is the highest completed education in the household? What is the total gross income before taxes of the household? On how many weekdays (Monday-Friday) was at least one person at home during the day (9 am – 4 pm) during an average working week in the period January - March 2021? Do you agree or disagree with the following statements? People who adjust their electricity consumption based on the price of electricity should be able to save on electricity costs. Do you usually actively follow your own electricity consumption? How do you get information about your electricity consumption? Do you actively follow how electricity prices vary from day to day and hour to hour?

**Table 6:** (*continued*)

Survey 3
How do you get information about electricity prices? Could any of the following motivate you to reduce your electricity consumption in hours of high electricity prices when it is cold outside and you are at home? Why do you not want to reduce your electricity consumption? Would you use a free information service that notifies you when the price of electricity the next day would be very high for a few hours? How much higher did the expected cost of electricity for the entire next day have to be for you to want to be notified?... Kroner higher compared to a normal winter day. Why do you not want to use such an information service? Imagine that there is a high electricity price some weekdays (Monday - Friday) in winter. How much money do you have to save on the electricity bill to reduce electricity consumption for a few hours in the middle of the day with a high electricity price? A few hours 1 weekday 1 winter month when the electricity price is high Imagine that there is a high electricity price some weekdays (Monday - Friday) in winter. How much money do you have to save on the electricity bill to reduce electricity consumption for a few hours in the middle of the day with a high electricity price? A few hours 5 weekdays 1 winter month when the electricity price is high Imagine that there is a high electricity price some weekdays (Monday - Friday) in winter. How much money do you have to save on the electricity bill to reduce electricity consumption for a few hours in the middle of the day with a high electricity price? A few hours all weekdays 1 winter month when the electricity price is high Imagine that there is a high electricity price for 2, 4, or 8 consecutive hours one weekday (Monday - Friday) in winter. How much money do you have to save on the electricity bill to reduce electricity consumption during these hours in the middle of the day with a high electricity price? 2 consecutive hours 1 weekday 1 winter month when the electricity price is high Imagine that there is a high electricity price for 2, 4, or 8 consecutive hours one weekday (Monday - Friday) in winter. How much money do you have to save on the electricity bill to reduce electricity consumption during these hours in the middle of the day with a high electricity price? 4 consecutive hours 1 weekday 1 winter month when the electricity price is high Imagine that there is a high electricity price for 2, 4, or 8 consecutive hours one weekday (Monday - Friday) in winter. How much money do you have to save on the electricity bill to reduce electricity consumption during these hours in the middle of the day with a high electricity price? 8 consecutive hours 1 weekday 1 winter month when the electricity price is high How much money do you need to save on your electricity bill to reduce your electricity consumption with the following measures? Reduce the indoor temperature by 3 degrees between 4 pm and 8 pm on 1 weekday in the winter while you are at home How much money do you need to save on your electricity bill to reduce your electricity consumption with the following measures? Do not charge the electric car at home, so you have to stop 1 hour to charge on the next long car trip Imagine that you buy smart equipment that will reduce your electricity bill by automatically shifting parts of your consumption away from hours with high electricity prices, and you notice nothing. How much do you have to save per year to buy such smart equipment that costs NOK 5,000? Imagine you could reduce your electricity bill by reducing your electricity consumption during hours of high electricity prices in winter. If you could choose, which of the following options would you prefer? Your household participated in Statnett's price experiment during the winter months. We are interested in how you experienced the experiment and have therefore some questions. Did you actively follow the price signals given during the experiment? Did you undertake measures in your household to reduce or shift electricity consumption during hours with high prices? What measures did you undertake? You earned money during the price experiment thanks to your measures. Do you think it was worth undertaking the measures? Why did you undertake the measures? Why did you not undertake such measures? Why did you not actively follow the price signals given during the experiment?

### 3.5 Outdoor temperature

The electricity consumption data were enriched with temperature data from the Norwegian Meteorological Institute [4], licensed under the Norwegian license for public data and Creative Commons 4.0 International (CC BY 4.0). For each region, one weather station was chosen to represent the temperature of the whole region, as described in Table 7. In addition to the hourly temperature series, the moving average of the past 24, 48, and 72 hours, including the actual hour, were calculated. These moving averages are provided since they may consider the thermal inertia of the buildings better than the actual hour value.

**Table 7:** Experiment regions and the respective weather stations with hourly outdoor temperature data.

Region	Station name	Station number	Height above mean sea level	Latitude	Longitude
Oslo	Oslo - Blindern	SN18700	94 m	59.9423° N	10.720° E
Stavanger	Stavanger - Våland	SN44640	72 m	58.9563° N	5.7278° E
Bergen	Bergen - Florida	SN50540	12 m	60.3830° N	5.3327° E
Trondheim	Trondheim - Risvollan	SN68230	84 m	63.3987° N	10.4228° E
Bodø	Bodø – Skivika	SN82310	5 m	67.3084° N	14.4309° E
Tromsø	Tromsø	SN90450	100 m	69.6537° N	18.9368° E

### 3.6 Structure of the table files

All data are stored in comma-separated text files, which are described in detail in the following sub-chapters:

• participants.csv
• experiment_days.csv
• data_hourly.csv
• price_signals.csv
• survey1_questions.csv, survey2_questions.csv, survey3_questions.csv
• survey1_answers.csv, survey2_answers.csv, survey3_answers.csv

In addition, all data are stored as objects in an RData file which can be directly imported into R, an open-source programming language for statistical analyses [1].

### 3.7 participants.csv

This file contains an overview of all participants in the project and their main characteristics, such as whether they were part of a treatment or control group, answered the surveys, and the household location. These data can be used to easily create sub-datasets by filtering the participants on specific characteristics. Each participant has a unique ID that connects the data with *data_hourly.csv* and the survey answers (*survey1_answers.csv, survey2_answers.csv, survey3_answers.csv*). A detailed description of the file structure is provided in Table 8.

**Table 8:** Description of the file participants.csv.

Column name	Possible values	Short description
ID	Exp_1, …, Exp_5410, Ipsos1, …, Ipsos2000	Unique ID for each household/participant
Participation_Experiment	Yes, No	Specifies if a household participated in the pricing experiment (*Yes*) or is only part of the survey panel (*No*)
Recruitment_organisation	Electricity supplier 1, Electricity supplier 2, Electricity supplier 3, Market research company, Transmission system operator	The organisation that recruited and communicated with the participants
Municipality	Asker, Bergen, Bodø, Bærum, Drammen, Lillestrøm, Oslo, Stavanger, Tromsø, Trondheim	Location of the household
Region	Bergen, Bodø, Oslo, Stavanger, Tromsø, Trondheim	Location of the household
Survey1_answered	Yes, No, NA	Specifies if a household has answered the survey, *NA*: non-participating households
Survey2_answered	Yes, No, NA	
Survey3_answered	Yes, No, NA	
Participation_Phase	Phase_1, Phase_2, Both	The household participated in the price experiment or the surveys in this project phase (*Phase_1*: winter 2019/20, *Phase_2*: winter 2020/21).
Control_Price_Phase1	Control_group, Price_group, NA	Specifies if a household was part of a control (*Control_group*) or a treatment group (*Price_group*) in the experiment in this phase, *NA*: non-participating households
Control_Price_Phase2	Control_group, Price_group, NA	
Group_Phase1	H1, H2, Control, NA	Specifies to which specific control or treatment group a household belonged in the experiment in this phase, *NA*: non-participating households
Group_Phase2	Bo_1, Bo_Control, Ber_1, Ber_Control, Os_1, …, Os_6, Os_Control, Trond_1, Trond_Control, Trom_1, Trom_2, Trom_Control, H1, H2, Control, NA	
Participation_status_Phase1 Participation_status_Phase2	OK, No_information, NA OK, No_information, NA	Status of the households in project hase 1 or 2. The data set includes electricity consumption data for all households with *OK* or *No_information*. *No_information*: withdrew their consent to get notice of pricing event days during the experiment. *NA*: non-participating households

### 3.8 experiment_days.csv

This file contains information about the experiment days of each treatment group. It summarises information extracted from *data_hourly.csv* to increase the understanding of the experimental setup. A detailed description of the file structure is provided in Table 9.

**Table 9:** Description of the file experiment_days.csv.

Column name	Possible values	Short description
Participation_Phase	Phase_1, Phase_2	Project phase the experiment day occurred (*Phase_1*: winter 2019/20, *Phase_2*: winter 2020/21)
Price_signal	A_5, A_10, B_2, B_5, B_10, B_15, C, P_2, P_5, P_10, P_15, P0_2, P0_10, P0_30	This price signal was used on the experiment day. The hourly price signals are described in *price_signals.csv*.
Date	yyyy-mm-dd	Date of the experiment day
Group	Bo_1, Ber_1, Os_1, …, Os_6, Trond_1, Trom_1, Trom_2, H1, H2	The treatment group which got the price signal on that experiment day

### 3.9 price_signals.csv

This file contains information about the price signals. Each price signal consists of 24 hourly prices over one day. A detailed description is provided in Table 10.

**Table 10:** Description of price_signals.csv.

Column name	Possible values	Short description
Price_signal	A_5, A_10, B_2, B_5, B_10, B_15, C, P_2, P_5, P_10, P_15, P0_2, P0_10, P0_30	Identifier for the different price signals. The letter identifies the price profile and the number of the price level in the peak price hours.
Price_profile	A, B, C, P, P0	Identifier for price profile
Price_level	2, 5, 10, 10/15, 15, 30	Price level in NOK/kWh in peak price hours. Profile C has two peak periods with prices of 10/15.
Hour	1, 2, …, 23, 24	Hour of the day
Experiment_price_NOK_kWh	0 – 30.15	Hourly experiment price in NOK/kWh

### 3.10 data_hourly.csv

This file contains all observations that change over time for each household, e.g., electricity consumption data, price signal, and outdoor temperature in the household's region. All observations have an hourly resolution. A detailed description is provided in Table 11. These data are only available for households that match all of the following conditions as specified in *participants.csv*:

• Participation_Experiment: Yes
• Participation_status_Phase1 or Participation_status_Phase2: OK, No_information

**Table 11:** Description of the file data_hourly.csv.

Column name	Possible values	Short description
ID	Exp_1, …, Exp_5410, Ipsos1, …, Ipsos2000	Unique ID for each household/participant
From	yyyy-mm-dd hh:00:00	Point in time, the measurement of the hourly electricity consumption starts
Date	yyyy-mm-dd	Date
Hour	1, 2, …, 23, 24	Hour of the day
Participation_Phase	Phase_1, Phase_2	Project phase of the price experiment (*Phase_1*: winter 2019/20, *Phase_2*: winter 2020/21)
Demand_kWh	0 – 22.26	Electricity consumption in kWh during that hour
Price_signal	A_5, A_10, B_2, B_5, B_10, B_15, C, P_2, P_5, P_10, P_15, P0_2, P0_10, P0_30, empty value	The price signal that the household got on that date. *Empty value*: no experiment day or household is not part of the control group
Experiment_price_NOK_kWh	0 – 30.15, NA	The experiment price in NOK/kWh for the specific hour and household. *NA*: no price signal was sent at that hour
Temperature	-21.8 – 12.8	Present value of air temperature 2 m above ground in °C from one specific weather station for each region
Temperature24	-18.1 – 10.6	Average temperature of the past 24 hours including the present hour
Temperature48	-17.3 – 9.7	Average temperature of the past 48 hours including the present hour
Temperature72	-17 – 9.1	Average temperature of the past 72 hours including the present hour.

### 3.11 survey1_questions.csv, survey2_questions.csv, survey3_questions.csv

The participating households answered three surveys during the project: Survey 1 before and Survey 2 after the pricing experiment in Phase 1, and Survey 3 after the pricing experiment in Phase 2. These files contain all the questions and the answer alternatives of the respective survey, both in the original Norwegian version and translated into English. A detailed description of the file structure is provided in Table 12.

**Table 12:** Description of the files survey1_questions.csv, survey2_questions.csv, and survey3_questions.csv.

Column name	Possible values	Short description
Question_ID	q1, …, husstand Aq1, …, Aq22 QI1, …, Q83	Unique identifier for each question in each survey
Question	Text	English translation of the question
Answer	Text or NA	English translation of the answer alternatives *NA*: the answer was numerical and not multiple choice
Not_included_english	x or NA	All questions/answers marked with *x* are not translated into English and not included in the answer files, *survey1_answers.csv, survey2_answers.csv, and survey3_answers.csv*. Usually, these are comments or free-text answers.
Sporsmal	Text	Original Norwegian version of the question
Svar	Text or NA	Original Norwegian version of the answer alternatives *NA*: the answer was numerical and not multiple choice

### 3.12 survey1_answers.csv, survey2_answers.csv, survey3_answers.csv

These data files contain the answers of all households that have answered at least one of the three surveys. A detailed descirption of the file structrure is provided in Table 13. An overview of which surveys a household has answered can be found in *participants.csv*.

**Table 13:** Description of the files survey1_answers.csv, survey2_answers.csv, and survey3_answers.csv.

Column name	Possible values	Short description
ID	Exp_1, …, Exp_5410, Ipsos1, …, Ipsos2000	ID for each household that has answered a survey
q1, …, husstand Aq1, …, Aq22 QI1, …, Q83	Answer alternatives, numeric, free text, NA, -	Answers of each respondent to the question with the same Question_ID as the column name. The question text can be found in *survey1_questions.csv, survey2_questions.csv*, and *survey3_questions.csv* *NA*: Respondent did not have that question; not applicable *- (minus)*: Respondent did not answer this question (if numerical or free text answer) or did not choose that option (if multiple choice answer). These observations must be removed from numerical variables if one wants to perform mathematical operations on these variables.

## 4 Experimental Design, Materials and Methods

The pricing experiments were conducted as randomised control trials [5,6]. Households had to opt-in to the project and were randomly assigned to either control or treatment groups, also referred to as price groups, since they were exposed to price signals. In total, 14 different price signals were tested. These price signals were in addition to the household's electricity tariff, usually variable tariffs tied to the hourly spot price of electricity, which were not affected by the experiment. If households responded, they got a monetary reward corresponding to the magnitude of their response and the price signals. The following chapters describe in detail all these aspects of the experimental design.

### 4.1 Participant recruitment

In Phase 1 of the project, households were recruited from three regions in Norway: Oslo, Bergen, and Stavanger. Based on contact information stored in the database of the market survey company Ipsos, households were recruited by this company by phone from December 2019 to February 2020. They had to opt in by completing several steps:

1. Accept the invitation and answer Survey 1 by telephone
2. If the survey was answered, the respective households got an email with more detailed project information and a hyperlink to a webpage where they had to give consent to the use of their data in the project
3. If consent was given: the respective households got information via a webpage on how they could access their already existing profile on the national datahub Elhub and grant direct access to their electricity consumption to the project

Households that finished this cumbersome process were rewarded with gift cards of NOK 100. In addition, they could win other gift cards with a value of up to NOK 10,000 if they were still part of the project at the end of Phase 1 to minimise dropout.

The invitation text and information given to households when recruited in Phase 1 were as follows when translated to English from Norwegian:

*In accordance with Norwegian legislation, we inform you that in order to conduct the research study, we require your consent to link the answers from the survey to your electricity data (from Elhub). The purpose is to establish a dataset for research to investigate whether variations in electricity prices affect the amount of electricity Norwegian households use. With this, Statnett will have the opportunity to find answers to questions such as what the most important factors are so that you can adapt your electricity consumption – whether it's control options, equipment, financial conditions, or other factors.*

*Some participants will also be randomly selected to take part in a scientific pricing experiment. Further information about this will be sent via email at the beginning of the new year. In the experiment, Statnett will test different prices and gain insight into how households react to them. The potential reactions from participants during the experiment may lead to financial gains.*

*Statnett will retain your personal information throughout the duration of the research project until April 1st, 2022. At the end of the project, Elhub access granted by participants to Statnett will be closed, ensuring that Statnett no longer has access. Participants in the research study will be informed via email once this access has been reset. Additionally, all data will be anonymised, making it impossible to identify individuals.*

*You always have the option to inform us if you wish to delete your personal information. If you would like further information in this regard, please visit the following page to learn more about the project and to find contact information.*

○ *Yes, I consent.*
○ *No, I do not consent.*

Additional information was given before participants shared their data from the national electricity database Elhub:

*In the access type field, select 'Full Access' and then press the 'Grant Access' button. By doing so, you consent that Statnett's research department can access metering data about your electricity consumption, as well as basic information about your grid company, your electricity provider, and your address. This information is crucial for the research project and will be analysed in an anonymised form. All personal data will be processed in accordance with GDPR regulations. You also have the right to withdraw your consent whenever you wish.*

In Phase 2, the households were recruited by their respective electricity supplier in November and December 2020. These companies sent invitations by email to their customers in selected cities: Oslo, Bergen, Trondheim, Bodø, and Tromsø. Each city was covered by one electricity supplier. Based on the experience from Phase 1, the recruiting process was designed to be as simple and user-friendly as possible. Participants could accept the invitation by consenting that their electricity consumption data could be shared with the project and that the project could contact them. As an incentive for participation, recruited households could win a reward. In addition, all participants from Phase 1 were informed that they still were part of Phase 2 and that they could opt out. All recruitment statistics with the rewards are summarised in Table 14.

**Table 14:** Summary of the participant recruitment and the final number of participating households.

Project phase	Recruited by	Households				
		Invited	Accepted invitation	Dropout/ exclusion	Final (% of invited)	Recruitment reward
*Phase 1*	*Market research company*	*22,000*	*894*	*152*	*742* *(3.4%)*	*Gift cards (once NOK 100, plus up to NOK 10,000 NOK)*
*Phase 2*		*35,793*	*5,077*	*702*	*4,375* *(12.2%)*	
	From Phase 1 (opt-out)	793	738	50	688 (86.8%)	None, all recruited in Phase 1
	Electricity supplier 1	15,000	3,001	539	2,462 (16.4%)	Two smartphones
	Electricity supplier 2	15,000	853	63	790 (5.3%)	Free electricity for one year
	Electricity supplier 3	5,000	485	50	435 (8.7%)	Free electricity for one year, football season ticket

The invitation text and information given to households when recruited in Phase 2 was as follows:

*By participating in the iFlex Project, you consent to receive messages about experimental electricity prices and to be contacted via phone or email for a survey.*

• *Yes, I consent.*

*Privacy: Participation in the iFlex Project*

*Participation in the project requires that data about your electricity consumption can be used and that you may be contacted at a later time to conduct a voluntary survey. All information about you will be processed in accordance with the EU General Data Protection Regulation (GDPR) and national privacy laws. Only we at your electricity company and the company conducting the survey will have access to your contact information (e.g., name, address, and phone number), making us the only ones capable of identifying you. Your consumption data, municipality number, and any survey responses will be shared in anonymised form with the iFlex research project for scientific analysis. The entire dataset without contact information or any other details that could directly identify you will be stored in a research data archive. This data will be made available for further use in scientific studies by researchers, students, and others interested in consumption data from Norwegian households. It should not be possible to re-identify you after the data is anonymised. However, for the sake of clarity, we want to inform you that re-identification could potentially occur if consumption data is combined with non-publicly available information from Elhub. This is illegal and should be considered highly unlikely. Access to your data from Elhub is granted only to organisations that have received your consent. Generally, these are companies with a customer relationship with you, such as your electricity provider and grid company. The results of the research project will be published in various channels, including scientific journals and the project's website. No participant can be identified in the results.*

*Your Rights as a Participant*

*As long as we can identify you, you have the right to withdraw your consent to data processing, to have any information we have about you changed or deleted, and to inquire about the information we hold about you. Once anonymised data has been shared, it's no longer possible to delete information, as it would require a significant effort to re-identify you using the remaining details. You also have the right to file a complaint with the Norwegian Data Protection Authority.*

### 4.2 Participant dropouts and exclusion

After their successful recruitment, households could be excluded or drop out later for several reasons, namely wrong recruitment, withdrawal from the project, missing data, and data outliers. Table 15 summarises why households were not part of the dataset after their successful recruitment in the two project phases.

**Table 15:** Overview of why recruited households are not part of the final dataset.

Project phase	Recruited by	Dropout/exclusion				
		Total number	Wrong recruitment	With-drawal	Missing data	Data outliers
*Phase 1*	*Market research company*	*152*	*96*	*0*	*48*	*8*
*Phase 2*		*702*	*9*	*86*	*577*	*30*
	Transmission system operator (opt-out)	50	0	25	25	0
	Electricity supplier 1	539	9	48	459	23
	Electricity supplier 2	63	0	13	47	3
	Electricity supplier 3	50	0	0	46	4

Wrong recruitment includes participants who did not adhere to the selection criteria of customer type and location and where it was difficult to find the correct electricity consumption meter. All recruited participants who were not households located in the predefined regions were excluded. This happened mainly in Phase 1 since the address information in the household database of the market survey company was not always up to date. For some recruited participants, matching one particular electricity consumption meter to the household was impossible, and they had to be excluded.

Withdrawal from the project was an option that all participants had at any point during the project. In Phase 1, no direct withdrawals of participants occurred. However, some participants used the possibility to withdraw their consent to get information from the project, including price signals and an invitation to surveys. However, their electricity consumption data were still collected and included in the final data set. In Phase 2, some participants withdraw from the project.

Missing data consist of households where electricity consumption data could not be collected for the entire project period. In Phase 1, some participants moved from their homes, so we lost the consent to collect data from the meter. Other households had missing data due to technical reasons, i.e., no data were available in the national electricity consumption database Elhub. In Phase 2, most missing data occurred because households moved their power contract to another electricity supplier during the project period. Data were incomplete for these households since the project had only access to electricity consumption data from the electricity suppliers that were partners in the project. This reason was not an issue for Phase 1 participants since the data were directly collected from the national database Elhub, regardless of the households' electricity suppliers. In addition, some participants were excluded in Phase 2 when they moved to a new home.

**Fig. 4:**
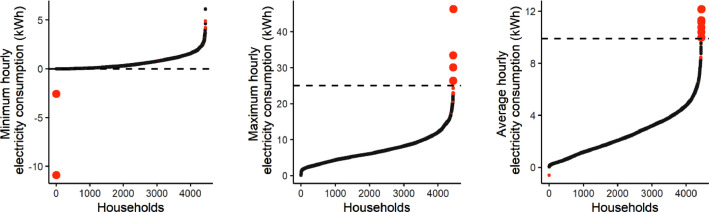
Minimum, maximum, and average hourly consumption of all households and the exclusion criteria (dashed line) for defining outliers (in red).

Data outliers include households with an electricity consumption profile that differs significantly from the rest of the sample. First, households with more than 99% of zero values in their electricity consumption data were excluded from the dataset. Secondly, three criteria were defined based on a graphical investigation of the minimum, maximum, and average hourly electricity consumption distribution to distinguish outliers from the rest of the household sample. Households with a minimum below zero, a maximum over 25 kWh, or an average over 9.9 kWh were excluded from the data set. Fig. 4 shows the graphical representation of these criteria and the excluded households.

### 4.3 Representativeness of the household sample

The representativeness of the household sample was a major concern of the participant recruitment, and self-selection bias led inevitably to differences between the population and the household sample. In general, the bias is larger for households recruited in Phase 1 than Phase 2.

In Phase 1, participating households had to fulfil a recruiting process with several steps and the need to log on to the Elhub website, which led to a low recruitment rate of 3.4% as described in section 4.1. These households are not representative of the general population, and the final sample had an overweight of households living in houses, with an age between 50 to 80 years, men, and couples without children. On the other hand, a clear underweight of households with apartments, with an age between 20 to 40 years, women and single households have been reported.

In Phase 2, surveys were sent to experiment participants and a representative sample of the population in the same regions to be able to compare main characteristics and socio-demographic variables and thus to reveal biases. The comparison reveals that the experiment households seem to be quite representative, and only minor differences and thus, biases to the general population exist as presented in Table 16.

**Table 16:** Comparison of selected household characteristics of experiment households and households sampled from the general population. Answers were weighted accordingly to the distribution of the experiment households over the different regions.

	Experiment households	Population	Difference
Answers	1,733	1,800	
Apartment	34%	40%	-6%
Living space in m^2^	119	110	9
Electric vehicle	18%	14%	4%
Direct electric heating	55%	57%	-2%
Heat pump	32%	26%	6%
Electric warm water boiler	76%	64%	12%
People per household	2.4	2.3	0.1
Household with children	25%	20%	5%
Low income	7%	9%	-2%

### 4.4 Group sample size

The minimum sample size was calculated upfront based on historical electricity consumption data from Norwegian households. The calculation resulted in a needed sample size of 180 or more households per group for detecting an electricity demand change of 5% in one hour with a significance level of 0.05 and statistical power of 0.8. Recruited households were randomly assigned to their respective group, and the study aimed at an initial group size of at least 220 to allow for dropouts of households during the project. In Phase 1, the priority was to reach a suitable sample size at the group level, not the region level, whereas, in Phase 2, the groups were defined per region. One exemption from the desired group size is the control group of Bergen in Phase 2, with only 52 participants. Due to the low number of recruited participants, it was decided to prioritise the treatment group. However, the number can be increased by including households from the control group ‘Control’ from Phase 1 who live in Bergen.

### 4.5 Price signals

The price signals were only active on some chosen experiment days during the winter. These days were chosen one week ahead by selecting the coldest day(s) based on the weather forecast for the different city regions to ensure much data from days with high electricity consumption, which is directly linked to heating in Norway. Each treatment group was exposed to one price signal chosen out of four predefined price signals. The respective price signal was chosen randomly but could also be changed to another one to ensure an approximate equal occurrence of all price signals per group.

Households in the treatment groups were informed via SMS or a push message one day before an experiment day. In project Phase 1, the information was sent at 3 pm, whereas it was sent at 6 pm during Phase 2. The message contained the following information: ‘*Hello! Tomorrow is an experiment day of the iFlex project. You can see the experiment prices and how you can earn money by adjusting your electricity consumption here: #Hyperlink to web page#’.* The reward calculation is explained in more detail in section 4.7.

**Fig. 5:**
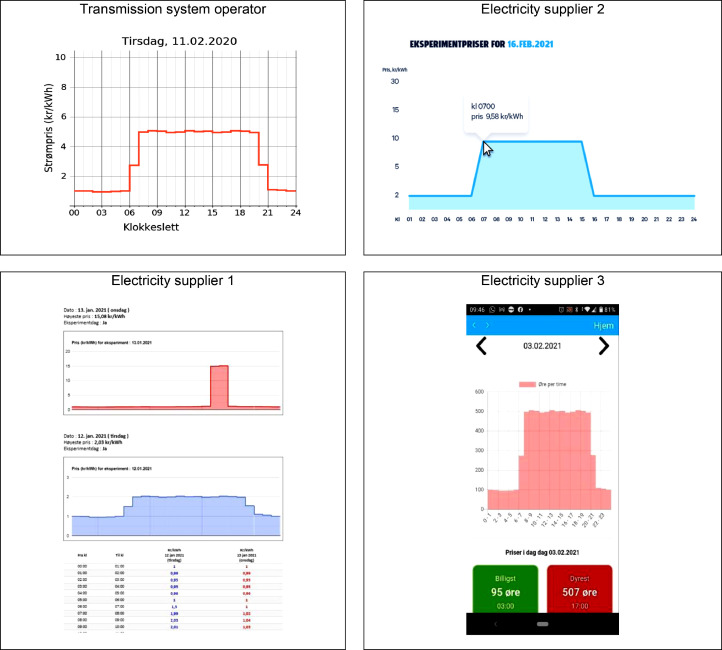
Examples of how the price signals were presented to the treatment groups via a webpage or an app dependent on the responsible project partner.

Each project partner with household participants had created a web page or an app with price charts. All web pages contained the same information but presented it differently. Fig. 5 shows how the project partners chose to visualise the price signals to their treatment groups.

### 4.6 Additional information for treatment groups

In contrast to the control groups, all households in the treatment groups got additional information on how they could reduce their electricity consumption during peak price hours. Descriptions and examples of possible measures were provided on the web pages with the price signals and were focused on the most significant contributors to Norwegian households' electricity consumption: electrical heating, warm water, and electric car charging. In addition, the examples included information about how much money a household could earn based on different peak prices and measures to allow for informed decisions when the households adjusted their electricity consumption. The households received no immediate feedback after the experiment days about their earned rewards due to their response to the price signals. The sum of rewards of each household was first calculated and then shared with the respective household at the end of the project.

### 4.7 Reward calculation and economic incentive

**Fig. 6:**
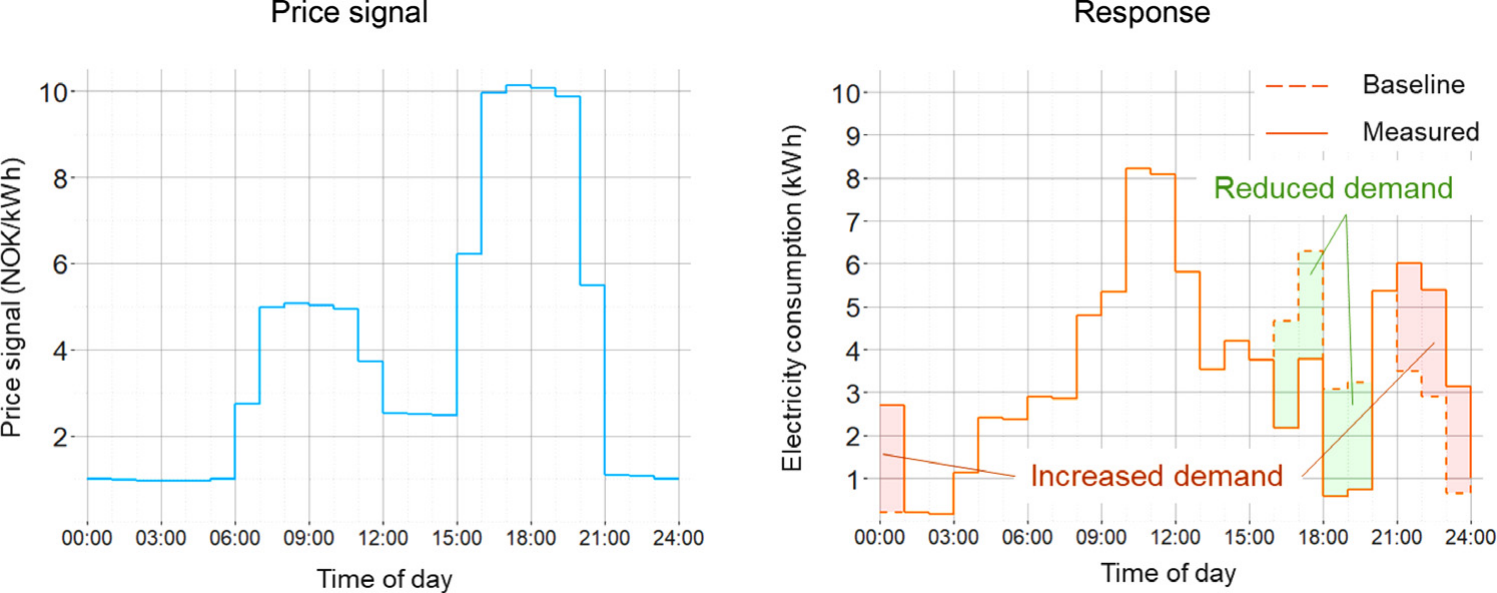
Example of a price signal and the imaginary response of a household with hourly reductions and increases in electricity consumption compared to the baseline. In this example, the reward would be around NOK 70.

The price signal did not replace the households' power contracts and grid tariff. If the households responded to the price signals, they could earn a reward based on the hourly price and their response. Calculating the counterfactual hourly electricity consumption without price signals for each household is necessary for estimating the response and reward. This baseline was calculated as the average electricity consumption of each hour of the day from the last ten non-experiment days, excluding weekends and holidays. In addition, the baseline was adjusted for systematic differences due to different weekdays and changes in outdoor temperature. All differences between the measured hourly electricity consumption and the baseline were regarded as a response from the household. This response was then multiplied with the hourly price signal to calculate the reward for the household. A household could also end up with a negative sum if it increased the electricity consumption in the peak price hours compared to the baseline. However, negative sums were zeroed out at the end, so no participant lost money. Fig. 6 shows an example of the imaginary response of a household on an experiment day with price signal C and the reward earned on this day.

After the price experiment was finished, the reward for all experiment days was paid to the households in two ways. All participants recruited by electricity suppliers got the final sum as a rebate on their electricity bill, whereas the other participants got a gift card. In Phase 1, the average sum was NOK 211 with a maximum of NOK 1,900, whereas the average response reward was NOK 154 with a maximum of NOK 3,600 in Phase 2.

### 4.8 Collection of electricity consumption values

The hourly electricity consumption data were collected via a smart meter that all Norwegian households have installed. The meter transmits the respective consumption data to the local grid companies and is stored in the national electricity database Elhub. Furthermore, Elhub redistributes the data to the electricity suppliers for billing purposes. The electricity consumption data in this dataset are collected directly from Elhub and the electricity suppliers for participants recruited by electricity suppliers.

### 4.9 Conduction of surveys

Three different surveys were prepared by the iFlex project and conducted with the help of the market research company Ipsos. In Phase 1 of the project, Survey 1 was sent before the pricing experiments started, whereas Survey 2 was distributed to the respondents after the pricing experiments. In Phase 2, the questions of surveys 1 and 2 were combined into Survey 3, and the households were asked to complete it after the pricing experiments. Based on the survey results from Phase 1, questions with limited relevance were discarded for Survey 3 to allow for a survey that could be answered in around 10 minutes.

The survey response rates of project participants were around 50%, as shown in Table 17. However, Survey 1 had a response rate of 100% since it was an integrative part of the recruitment process via telephone, and participants had to answer before they could participate in the project. If one takes the contacted households as a basis, the response rate would be only 8%, including households not interested in participating in the project.

**Table 17:** Survey methods and response rates.

	Project phase	Conduction period	Invitation method	Survey method	Population	Response rate
Survey 1	Phase 1	10 December 2019 - 10 March 2020	Telephone	Telephone	Participants Phase 1	100 % (8 %)
Survey 2	Phase 1	12 May - 9 June 2020	Email with hyperlink	Web	Participants Phase 1	52 %
Survey 3 - Participants	Phase 2	12 May - 10 June 2021	Email with hyperlink	Web	Participants Phase 2	47 %
Survey 3 – Norway	Phase 2	12 May - 10 June 2021	Email with hyperlink	Web	Pre-recruited Ipsos-panel	Not known

## Ethics Statements

All participants gave informed consent to share their electricity consumption data and to allow for a combination with the survey answers so that the anonymised data could be published. The data were anonymised by removing all personal data and aggregating address information into municipalities, the smallest being Bodø with 24,000 households.

It exists a risk of re-identification of the households in the dataset by using the electricity consumption data and comparing them with the data stored in Elhub. This is illegal and should be considered highly unlikely. Only organisations that have received the consent of the household have access to this kind of data from Elhub. Generally, these are companies with a customer relationship with the household, such as their electricity provider or grid company. Furthermore, electricity consumption data older than three years are automatically deleted in Elhub, ensuring that the possibility of re-identification should be even lower after around April 2024.

## Declaration of Generative AI and AI-Assisted Technologies in the Writing Process

During the preparation of this work the authors used ChatGPT and Grammarly to translate the invitation to the project and the consent from Norwegian to English, and to improve readability and language in general. After using these tools, the authors reviewed and edited the content as needed and take full responsibility for the content of the publication.

## CRediT authorship contribution statement

**Matthias Hofmann:** Conceptualization, Methodology, Investigation, Data curation, Writing – original draft, Writing – review & editing, Visualization, Supervision, Project administration, Funding acquisition. **Turid Siebenbrunner:** Supervision, Project administration, Writing – review & editing.

## Data Availability

A rich dataset of hourly residential electricity consumption data and survey answers from the iFlex dynamic pricing experiment (Original data) (Zenodo)
